# Negative manifestations of internalized extreme filial piety culture among Korean high school student athletes: dropping out, “Athlete Melt”, tie-in sale, and “Running Away”

**DOI:** 10.3389/fspor.2025.1558398

**Published:** 2025-08-04

**Authors:** SangJin Yoon, SeaMi Lim

**Affiliations:** ^1^ Sport Coaching Science, Graduate School of Health and Sport Science, Nippon Sport Science University, Tokyo, Japan; ^2^ The Department of Sport Science, The College of Arts and Physical Education, Incheon National University, Arts & Physical Education College, Yeonsu-gu, Incheon, Republic of Korea

**Keywords:** Korea, student-athletes, filial piety culture, dropping out, tie-in sale, running away, career dilemmas, athlete transition

## Abstract

**Introduction:**

This study investigates the adverse impact of extreme filial piety culture on the career trajectories of elite Korean amateur athletes following professional league contract failures.

**Methods:**

Using narrative interviews and participant observation, we explored the experiences of high school student-athletes who were unable to transition to professional sports, secure university admission as athletes, or chose to abandon their athletic careers.

**Results:**

Our analysis revealed four key phenomena: some student-athletes drop out of high school or forgo university to pursue other careers; illicit “tie-in sales” allow parents to leverage sports performance for admission to prestigious universities; several student-athletes run away from home due to abusive or unsupportive environments and feelings of entrapment; and internalized extreme filial piety generates overwhelming guilt as athletes feel compelled to repay parental sacrifices through their success.

**Discussion:**

These findings highlight the need for policy interventions and support systems that broaden career options, curb excessive parental and coaching pressures, and foster greater autonomy in student-athletes’ decision-making.

## 1 Introduction

In Korea, parents play a pivotal, and often demanding, role in the journey of student athletes aspiring to become professionals (1–3). Parents influence their children's entry into sports (4–7) and shape their career as athletes (7–9). Particularly, the parents' involvement with their children's sports team will affect the child's inclusion in competitions; specifically, parents are expected to enter into personal relationships with the coaches and provide them with financial and other benefits (1, 10, 11). Parents frequently provide various forms of support, such as preparing food, purchasing training equipment, and coordinating team cheering. Additionally, they may engage in social interactions with coaches, including shared meals, karaoke outings, or drinking alcohol with the coaches after a game or practise. Sports requires parental support, both materially and emotionally (7, 12–15), and they inevitably end up overextending themselves by going above and beyond to provide for their children, such as buying expensive exercise equipment, providing assistance with personal training, or arranging private tutoring from professional athletes (2, 16–19). In the past, it was possible for children to participate in sports without parental involvement or support. However, sports programs today are increasingly privatized, affected by residential and living areas, becoming more expensive, and have complicated participation schedules and organizational structures (20). The privatization of sports programs has led to a growing dependence on parental involvement and financial support, making independent participation increasingly challenging for student athletes (3, 7, 21–27).

Although parental and coaching support play a crucial role, statistical data indicate that only 5%–10% of student athletes successfully transition to professional leagues in Korea (28–30). This discrepancy highlights the need for alternative career pathways and policies (30–35). This results in a typical elite sports pyramid where only a select few succeed professionally, while others must abandon their aspirations due to injury or insufficient skill (32, 35–39).

Most Korean student athletes in school sports departments dedicate their elementary, middle, and high school years solely to training, often at the expense of academics (3, 40–42); they undergo life experiences that differ from other students (35, 43–45). This is because student athletes believe that they can succeed as an athlete, and sacrifice the potential future rewards of excellent grades (e.g., college admissions, professional admissions, financial rewards) in exchange for the reality of being forced to overtrain and forfeiting their right to learn (30, 40, 46, 47).

Thus, if they leave sports, they face significant challenges due to having focused solely on athletics, often sacrificing even fundamental education at an early age (17, 48, 49). As a result, finding alternative career paths becomes difficult once they recognize their inability to go professional (1, 2, 50, 51).

The success rate for athletes is statistically very low (29, 52), and despite many student athletes quitting or even running away from home to escape sports, the number of aspiring athletes in Korea remains high (3, 35, 53–55). This phenomenon may stem from strong parental enthusiasm for education (56), high parental aspirations for their children's success (57), and deeply ingrained social expectations of parental dedication (58, 59). Previous studies have examined the role of parents in athletic socialization, but research into why some parents persistently support their children's athletic careers remains limited (5).

Although most student athletes in Korea's school sports departments deal with unfavourable circumstances, and some even experience the need to run away from home to escape the pressures of sports, research on why certain parents continue to push their children toward athletic careers remains insufficient. To address this gap, understanding the culture of school sports departments and the role of parents within this environment is crucial.

To that end, this study explored the emotions and influences experienced by student athletes in the course of their parents' sports parenting behaviour. It further analyzes the experiences of student athletes in school sports departments, focusing on the influence of extreme Confucian filial piety, which remains prevalent in Korean society (60, 61).

Filial piety culture involves respecting and caring for one's parents, obeying and supporting them, and refusing to engage in behaviour that humiliates the family (62, 63, 184). It particularly emphasizes the concept of family honour (64, 65). In Eastern societies, this cultural principle fosters a strong hierarchical relationship between parents and children (61, 63, 65–67).

Filial culture is very interactive and entails the child reciprocating parental sacrifices (68–71). It is ubiquitous, although there are terminological differences worldwide, and the depth of relationships may vary due to differences in religion, region, and era (60, 68, 72–74). Confucianism believes that “filial piety is the foundation of virtue”. Similarly, in Christianity, predominantly representative of the West, one of the 10 Commandments in Chapter 20 of the Book of Exodus states, “Honor your father and your mother” (70, 71).

Filial piety has been recognized as the highest virtue in Confucian countries such as Korea, China, and Japan for thousands of years (69, 75–78). As such, Eastern Confucianist filial piety culture is constantly being recreated throughout history and social contexts (62, 79–82, 185), and it has changed directions according to changes in historical experience, political governance, and socioeconomic structure (62, 80, 82–86).

In China, the Confucian traditional ideology of the family was prohibited as counter-revolutionary after the socialist revolution in 1949, and the onus of community responsibility was placed on the political leader (87, 88), thus weakening the culture of filial piety in China (61, 79, 89).

In Japan, the tradition of oriental filial piety continued pre-World War II, and the *ie* system or patriarchal immediate family system was created (90, 91), which was abolished in 1948 (91, 92). Subsequent decreases in the children's tendency to support their parents has led to a weakened family support consciousness (61, 78, 91).

In contrast, in Korea, which has long adhered to the Confucian culture, there is a rare deep parent-child bond (74, 93). Evident from the Act Regarding the Encouragement and Support of the Practice of Filial Piety, intended for a level of long-term conservation and encouragement of the filial piety culture not witnessed elsewhere, Koreans deem filial piety to be crucial and respond sensitively to it (60, 71, 93, 94).

As most adolescents in Korea receive unconditional love and attention from their parents and grandparents from an early age, they do their best to meet the expectations of their families in return, even if their parents do not want repayment (60, 83, 93, 94). This is a manifestation of extreme filial piety; where parents blindly sacrifice for their children, and the children feel that they owe their parents a debt that they cannot repay (74, 83, 94). Korea's distinctive history and social context brought about this extreme version of the Oriental Confucian filial piety culture.

Especially, this phenomenon manifests more intensely among student athletes. The minute someone becomes an athlete at the behest of respected elders like a coach or parents or their own desires and improves competency to a certain extent, they fall into the fetters of extreme filial piety culture (83). As expectations of success as a professional athlete increase, the burden on both the parents and the child increases (6, 95). This is because the failures of children are perceived as the failures of parents in Korean society (83, 96–98).

In Korea, if the child has a good job, the parents think that their child's social status and treatment will be applied equally to themselves. Even if the parent's social status is low, if their child's job entails a high social status, they think their own social status will rise as a result (99–101). The dream is to raise the status within the generations through elevations in status between generations, especially in low-income households (101–107).

In pursuit of their children's success, many Korean parents make significant financial and emotional sacrifices, often going beyond what is typically expected (3, 13, 71, 108–110). They engage in extensive support activities (21, 108), including ingratiating themselves with coaches and contributing financially to the team (111, 112). However, as only a small fraction of student athletes achieves professional status (113), the majority face an abrupt transition out of sports, often accompanied by intense feelings of guilt and frustration. This burden is exacerbated in low-income households, where sports are seen as a potential path to social mobility (42, 105). When success does not materialize, both parents and children struggle with the emotional and financial consequences of their deep investment, reinforcing the pressures of extreme filial piety (96, 102, 103, 106, 107, 114, 115).

On the above basis, this study examines how the internalization of extreme filial piety influences the career trajectories and decision-making processes of Korean student athletes. Despite the slim chances of achieving professional success, many athletes persist due to deeply ingrained familial and societal expectations. The research aims to identify the psychological and social difficulties faced by student athletes when they fail to meet these expectations, particularly focusing on issues such as dropping out, “athlete melt”, tie-in sales, and running away. Through in-depth interviews, this study explores the mechanisms by which extreme filial piety shapes both parental and student behaviors, revealing how it reinforces rigid career paths and exacerbates emotional distress. The findings contribute to the understanding of how cultural values affect athletic careers and suggest policy measures to provide more diverse and realistic career options for student athletes.

## 2 Methods

### 2.1 Participants

Participants were carefully recruited to represent typical examples of the topic of this study. To ensure confidentiality, pseudonyms were used, and all interviews were conducted with strict data protection measures. Given the study's nature, snowball sampling was utilized, with initial participants introduced through athletes from various sports with whom rapport had already been built. During this process, the researcher ensured personal protection, data confidentiality, and voluntary participation. The study's purpose was explained, and informed consent was obtained. The study focused on high school student athletes whose parents played a key role in their athletic careers or provided significant parental support. Additionally, it explored whether they had dropped out of high school or chosen not to enroll in university. A total of 11 individuals expressed interest in participating; however, two withdrew, leaving nine final participants. Additionally, Yang, a 36-year-old male tennis coach with a university degree, joined as an auxiliary participant at his own request. All participants actively agreed to in-depth interviews.

In total, nine high school athletes participated in the study, competing at the national and regional levels in Korea across five sports: baseball, basketball, soccer, tennis, and wrestling. Among the student athletes, two had dropped out of high school, three were currently attending university, and four had graduated from university. The group consisted of seven male and three female athletes. The participants were recruited from two major cities in Korea: Seoul and Busan. These cities were selected due to their high population density and diverse range of sports programs, ensuring a representative sample of student athletes.

Table 1 presents the demographic and athletic characteristics of the student athletes, including their education level, sport, and gender. The study was conducted in accordance with the Declaration of Helsinki and approved by the Ethics Committee of Korea Institute of Sport Science (protocol code KISS-22038-2303-03).

**Table 1:** Personal characteristics of study participants.

No.	Name*	Age	Gender	Education level	Event
1	Seong	24	Male	High school dropout	Baseball
2	Kang	22	Female	High school dropout	Tennis
3	Yoon	24	Male	Attending University	Basketball
4	Lee	25	Male	Attending University	Wrestling
5	Kim	23	Male	Attending University	Soccer
6	Han	29	Female	University graduate	Tennis
7	Jeong	27	Male	University graduate	Soccer
8	Park	28	Male	University graduate	Baseball
9	An	28	Female	University graduate	Basketball
10	Yang	36	Male	University graduate	Tennis coach

* Pseudonyms have been applied to protect the identities of the study participants.

### 2.2 Data collection method

#### 2.2.1 In-depth interview

In-depth interviews were conducted with a focus on four study participants who had built a rapport with the researcher and actively participated in the research; oral consent was obtained from them. One unstructured interview was conducted with each of them for about 1 to 2 h, depending on participant availability. Subsequently, all study participants were asked questions related to their experiences in school sports departments, their relationships with parents, and personal reflections. The initial four unstructured interviews served as exploratory interviews that helped identify key thematic areas. These early interviews informed the refinement of subsequent interview strategies. Based on these findings, the researcher conducted semi-structured interviews with all participants, using open-ended questions to ensure depth while maintaining flexibility. The main topics explored were: parental involvement in athletic careers; decisions around school and sport; and the emotional burden associated with filial expectations.

The researcher made efforts to exclude those who felt even slightly burdened by the interview or did not express their active willingness to participate. The participants were informed about their ability to stop the interview at any given time.

Participants responded to the interview without significant difficulties, and the recorded data were transcribed. Interviews continued until data saturation was reached. Saturation was defined as the point at which no new themes or perspectives emerged from subsequent interviews. This was assessed through ongoing transcript reviews and comparison across cases, with recruitment ending when new data became redundant.

After scheduling interviews at locations chosen by participants and obtaining their consent, each participant took part in one or two in-depth, one-on-one interviews.

#### 2.2.2 Collection of auxiliary research data

Auxiliary research data were collected using search engines, such as Google and Naver, including the Korean academic database RISS4U. The collected research data were discussed in depth by advisors, including the researcher, and only the data deemed to bear sufficient significance were used in this study.

#### 2.2.3 Data analysis

This ethnographic study involved iterative reading, interpretation, and categorization of the collected qualitative data (116). Audio recordings made during fieldwork were transcribed for analysis. Thematic analysis was carried out using an inductive coding approach, without applying any predefined frameworks (117). All transcripts were reviewed multiple times to ensure familiarity with the data. Open coding was used to identify recurring patterns and meaningful units of text, which were subsequently grouped into higher-order categories and overarching themes through an iterative comparison process. Internal supporting materials (e.g., interview transcripts, field notes, and memos) were organized by the research team to ensure consistency and traceability throughout the analysis but are not publicly available.

#### 2.2.4 Reflexivity and positionality

As qualitative researchers, we are aware that our interpretation of participants' experiences is not value-neutral. Our role in selecting, framing, and interpreting the data inherently involves subjectivity. Throughout the research process, we remained reflexively aware of how our perspectives, assumptions, and interactions with participants could shape the research outcomes. This reflexive stance was essential to maintaining analytical transparency and ensuring that findings reflect both participants' voices and the interpretive context in which they were analyzed.

#### 2.2.5 Verification of data authenticity

Thick description is a method that provides overall information about the subject of description and describes an experience with context, intention, and meaning to allow readers to fully understand the situation (118). Therefore, the researcher did not report simple facts of all situations and actions that occurred to the study participants regardless of intention or context. Efforts were made toward in-depth descriptions, starting from experiences in school sports departments and the context, intention, and meaning of subsequent research results.

Additionally, efforts were made to eliminate distortions and errors that might have disrupted the analysis and interpretation of interview data.

## 3 Results and discussions

### 3.1 Dropping out and “Athlete Melt”^1^: the interplay between parental expectations and student-athlete decisions

Trow (119) classified stages of higher education development, indicating that if the university age group exceeds 50%, a stage of universalization has been reached. In Korea, the university enrollment rate reached 51.4% in 1995, and more than 70% of high school students become university students unless they face ongoing financial crisis (120, 121).

Similarly, Korean student-athletes generally aspire to attend university unless they transition directly into professional leagues. Because student-athletes from elementary to middle school dedicate themselves entirely to sports, often sacrificing academic studies, they seek to enter university through the sports specialist admission system^2^ (30, 97, 98, 122–128). However, as they approach university admission, both student-athletes and their parents face a critical dilemma. Some athletes, realizing that their chances of a successful sports career are slim, choose to drop out or experience “athlete melt”. This is often an attempt to ease their parents' financial burden, influenced by deep-rooted filial expectations. However, this decision often conflicts with parents' firm belief that their child's success reflects their own sacrifices and efforts. Many parents view their sacrifices as part of their duty, making it difficult for student-athletes to step away from sports without feeling a sense of failure—not only for themselves but for their families as well. This paradoxical tension between parental expectations and individual reality leads to a difficult and emotionally painful decision-making process. These outcomes—dropping out or experiencing athlete melt—result from either the student-athlete's decision being respected or parental acquiescence.

This case illustrates how parental expectations impose psychological and financial burdens on student-athletes, significantly influencing their dropout decisions.

It's so difficult to take care of athletes, and I felt guilty because I thought I wasn't doing well, so I wanted to reduce the burden on my parents. In my case, I have many family members and older sisters, so I tried my best to lighten their burden as much as possible … (sentence omitted) So I dropped out of school … (sentence omitted) Anyway, I thought it was too late to study now. I believed that if I succeeded as an athlete, studying wouldn't be necessary … (sentence omitted) I just didn't know that I wouldn't succeed as an athlete. (Kang, 22, female/tennis)

This account illustrates how student-athletes experience significant pressure due to their families' expectations, leading to decisions shaped by both personal aspirations and parental influence (6).

While financial concerns are important, student-athletes mainly believe that academics will not help their future as athletes. This perception strongly influences their decision to drop out or skip university (129, 130). According to Sun and Baek (42), 73.3% of high school student athletes stated that while they attended school, but did not actively participate in academic activities because they believed school classes were irrelevant to their future careers. Although school attendance is mandatory, the intense demands of athletic training often prevent student-athletes from engaging in academics (131). This highlights a systemic imbalance between education and sports commitments, particularly in the context of dual careers (30, 33, 35, 42, 132–135, 182). As a result, these athletes often lead highly specialized lives focused solely on sports, missing out on fundamental educational and social experiences (34, 131, 133).

Once student-athletes fall behind academically, it is almost impossible to recover while continuing their athletic training (136–138). If they stop playing sports in high school, their low GPAs prevent them from taking the KSAT college entrance exam^3^, severely limiting their chances of university admission. Additionally, those who fail to advance to professional sports teams lack both the academic ability to enter college and the athletic skills required for sports specialist admissions.

As a result, parents play a decisive role in shaping student-athletes' academic and career decisions (139, 140). Without such intervention, many student-athletes end up taking physically demanding jobs. In Korea, individuals whose highest level of education is high school graduation face implicit social stigma (106, 141–148). This social pressure pushes student-athletes and their families toward university, even when it doesn't match the athlete's goals or academic ability (130, 138).

Those who forgo university education face significant disadvantages in Korean society, with high school dropouts experiencing even harsher conditions (186–188). Even for part-time jobs, education level is often a required field on job applications. Although most student-athletes choose not to enroll in university rather than dropping out of high school, societal neglect and discrimination leave those who do not pursue higher education struggling for recognition as full members of society (106, 141, 142, 186, 187).

Therefore, while financial concerns are a factor, the primary reasons for dropping out or not enrolling in university lie in the structural issues of Korea's sports education system and the societal disadvantages faced by those without higher education (106, 147, 148).

In Korea, unless athletes are signed by professional teams, they must attend college to continue competing in sports. However, prestigious universities only admit top-tier athletes, leaving those with weaker sports records unable to continue their athletic careers. Student-athletes with lower athletic performance can still enroll in local colleges or junior colleges struggling with student recruitment, as these institutions often accept athletes without requiring the sports specialist admission system or entrance exam (149, 150).

However, for student-athletes with poor athletic ability aiming for a prestigious university, financially motivated admissions irregularities have been reported. A newspaper article reported that such under-the-table admissions require payments ranging from three to six times the annual salary of a typical office worker (151). Even these limited opportunities are available only to a select few.

I was completely at a loss. I would wake up early to get ready to go to my morning group training, but I felt an overwhelming emptiness on the first day after withdrawing from sports … (sentence omitted) I broke down in tears, feeling so small. I couldn't bring myself to face my parents in the room adjacent to mine, so I stayed in my room all day without eating. (Seong, 24, male/baseball)

Dropping out of high school or skipping university puts student-athletes and their families at risk of socioeconomic marginalization. During family gatherings, parents often feel a deep sense of shame and try to avoid discussing their child's future, while also struggling to respond when acquaintances inquire about their children's current pursuits (97, 98). This is because, in Korean society, the parents' sacrifices and their child's achievements are deeply interconnected, reinforcing extreme filial expectations.

Student-athletes often hesitate to express their wish to quit sports due to family expectations. Many delay action until external factors force them to change course. The structural reliance on parental support in elite sports further complicates this decision-making process (3, 21).

Meanwhile, a case in which a Korean athlete's father committed suicide in 2013 regarding a Taekwondo referee corruption was treated as an example of unfairness and unethical issues in the sports community (18), and it is also an example of frustration wherein the dedicated efforts and support from athlete's parents came to no avail due to referee corruption.

This illustrates a case in which factors other than a player's athletic ability were involved in the sports community, and it is also a case where bribes were paid by the parents or coaches of the opposing team. This is illustrative of why student athletes have no choice but to depend on their parents, a phenomenon that is also an example of student athletes and their parents becoming entangled in the culture of extreme filial piety.

Furthermore, parents' interest in the elite athletes' participation in sports is significant, as exemplified by soccer players Park Jisung and Son Heungmin, and golfer Park Seri's fathers and figure skater Kim Yuna's mother. The process of extreme dedication from such star athletes' parents often becomes intense and demanding. Parents’ intense sacrifices to help their children enter good colleges or find better jobs often make student-athletes feel obligated to succeed. This success is seen as a form of repayment for parental dedication.

For the parents of athletes, the success of their children is no different from their own success. To them, it's also a way for the child to give back. That's A form of filial piety, isn't it? Parents stake everything for their children's success, and that caused anxiety disorder in the case of OOO. I kept thinking, “If my parents sacrificed this much, then I have to succeed just as much”. That pressure was too much for me. I couldn't handle it anymore. so I ended up dropping out of school … (Yang, 36, male/tennis coach)

I felt deeply apologetic to my mother about going to university. She had a hard time. She even took on the role of general manager at the team parents' association because she was worried, I might feel discouraged for not being good enough. That was the hardest part. By the time I reached high school, I realized that my parents had to pay a lot of money for me to attend university. You come to understand this once you're in high school. In particular, it's difficult to enroll in prestigious universities in popular sports like mine. I could sense my parents' concerns. So, I was the one who first brought up the idea of dropping out. I told them I would quit sports and leave school … (Seong, 24, male/baseball)

Most of the study participants who responded to the interview felt apologetic toward their parents. This feeling may have arisen due to the excessively competitive environment of Korean sports along with the parents' purpose of supporting children. There is an underlying mentality of prioritising winning and ranking first, which may be noted as one of the most representative issues in the Korean sports community. Amid fierce competition, it has been demonstrated that the parents' commitment mentally pressures student athletes to “repay” them. Further, the causes of conflict appear not owing simply to the parents' expectations and greed but also to their sense of responsibility for guiding their children in the right way (152).

### 3.2 Tie-in sale: coerced compliance and its psychological consequences

A tie-in sale means bundling poor-quality items with desirable ones. It is used to get rid of low-demand products by attaching them to popular ones (153). Such tie-in sales have been employed by parents and coaches as a means to manipulate the career trajectories of student athletes (124, 154–156).

Sports specialist selection in Korean universities involves a structure wherein a university's main coach makes the first recommendation for players who meet the conditions and then makes a final decision through the selection committee (149). As selection committee members often lack specialized knowledge about the university's player requirements or the status of recruited athletes, the coach's initial recommendation holds decisive influence over college admissions (124, 149). In addition, when Korean high school players enter college, it is customary that they go through the high school and college coaches as intermediaries (41, 149). The high school coach arranges for the college to accept not only star player A, but also lower-skilled players B and C from the same school. Now the parents of players B and C must hold up their end of the deal and pay player A and the university manager, etc., instead of the university, in the name of player A's scouting expenses (157–159).

Although such practices are clearly prohibited under Korean law—specifically the Act on the Aggravated Punishment, etc. of Specific Crimes (160)—the tie-in sale strategy continues to exist. It is sustained through under-the-table payments made by parents of less-skilled athletes to university officials, high school coaches, or even top athletes' parents (41, 124, 149).

This practice allows student-athletes with lower athletic performance—who do not meet the criteria for sports specialist admissions or regular university entrance—to gain admission through corrupt recruitment.

In summary, these outcomes—exemplified by tie-in sales—arise when parental will be decisively imposed and student-athletes, by acquiescing to such practices, inadvertently perpetuate a corrupt recruitment system.

It used to be somewhat common. In particular, OOO had talents far too great for K University, making it feel like a waste. As far as I know, OOO told his/her parents in advance that he/she would enroll in K University on the condition that his/her younger sibling would also be admitted to K University. Whether this was a sacrifice is debatable, the costs of raising him/her were offset by helping a younger sibling. (Yang, 36, male/tennis coach)

Considering the money my parents spent, the coach had no right to say anything. Large bribes were given, team dinners were paid for, and even after I graduated, I still covered team dinner expenses. Despite that, they asked me for money if I wanted to go with OOO, one of the key players, to T University. I said I wouldn't go because the whole situation felt corrupt. But things don’t work out that way—somehow, my parents managed to gather the money to pay (sentence omitted) I feel deeply apologetic toward my parents. (Han 29, female/tennis)

For me, I've seen kids who got into university that way and then moved on to business teams the same way, coerced by coaches into attending mid-tier universities because they were blocked from enrolling in higher-ranking ones. It's ridiculous. I clearly told the manager I didn't want to join that team, but the contract had already been signed. I was so angry that I argued, but they said my parents did it. My father had signed the contract. What more could I say? I still don't like it. I'll transfer to another business team as soon as my contract expires. (Lee, 25, male/wrestling).

My parents decided which university I would attend. The coach asked me in advance, but in the end, I couldn't go to the university I wanted. My parents were consulted, and my father made the final decision … (Yoon, 24, male/basketball).

As shown in the interviews, student-athletes have little control over decisions about their own careers (97, 98, 149, 161). Of course, devoted parents may discover their children's talents in adolescence and build follow-up plans out of the resulting hope (162, 163). Ultimately, student athletes who never practiced 'self-determination' in their adolescence end up leaving decisions regarding future careers entirely to their parents (163–165).

I felt both deeply apologetic and grateful that my parents gave up and sacrificed a lot for me. However, I also felt a lot of pressure. I thought I had to succeed, but that's not easy. Whenever I performed poorly in a game, I felt completely devastated and dreaded going home. I didn't know what I would do if I didn't become a professional athlete, especially after they invested everything in me. (Jeong, 27, male/soccer).

On days when I didn't get any base hits, the atmosphere in my house felt as if someone had passed away. It was unbearable. I didn't even want to go home. It was even worse in my senior year of high school. My chances of being recruited by a university or a professional team were all tied to that one moment. (Park, 28, male/baseball)

As seen in the study participant's statement, when student-athletes meet parental expectations, their actions are perceived as fulfilling filial duty; however, failing to do so can lead to significant psychological distress, including lower self-esteem and familial conflict.

Previous studies suggest that excessive parental involvement fosters a strong emphasis on education (56), an instrumental approach to achievement (58), and a deep identification with parental roles (59). However, the direct subjects of tie-in sales due to parental involvement suffer considerable stress and low self-esteem.

Honestly, I had wanted to quit sports for a long time because I'm not confident in those terms. One mistake, and I knew without a doubt that I would face verbal abuse from the coach. I hated everything about it and wanted to walk away, but I couldn't say it when I thought about my parents who had worked hard, taking care of me. For four years, I wrestled with this decision and brought it up a few times. But my parents always asked me, “What will you do if you drop out of university?” They told me there was nothing else for me and urged me to stick with it. I couldn't quit. I felt trapped—like if I quit, I would become nothing. (Kim, 23, male/soccer)

In Korean society, where the mere admission into a university located in the country's capital Seoul is a source of pride, the fact that someone could not enroll in a university, or had to enroll in a less prestigious university, is perceived as “unfilial” on the child's part and as a “failure” on the parents' part or in turn “unfilial” to the students' grandparents (71, 149). This uniquely Korean phenomenon arises from intense parental devotion. Parents often invest everything into their children's success. As a result, children feel obligated to “repay” them—often at the expense of their own self-esteem.

### 3.3 Running away from home: a silent clamour against an inescapable reality

Student athletes who responded to interviews could not easily answer the question about what they wanted to be other than athletes. The reasons may be that they were often unaware of what they could do and were ignorant about vocational options. As mentioned above, career paths for student athletes are often determined by their parents (124, 139, 140, 149, 154–156, 166), and once they start playing sports, because they also give up their academic studies, they have no choice but to proactively and actively cling to sports. Thus, there may be fears that student athletes will become social failures if they give up on sports without becoming professional players (30, 123, 125, 126, 128).

Furthermore, they are trapped in situations where they cannot easily give up sports even if they want to, due to other's expectations, parents' sacrifices, and coercion by coaches (30, 149, 167). As a result, some student-athletes run away from home—at least temporarily—to escape their hopeless situations (74).

This outcome—running away from home—results from an uncompromising standoff between parents and student-athletes, often leaving young athletes feeling trapped with no alternative but to escape. But running away rarely solves the problem. Most athletes return home with guilt and uncertainty about what comes next.

At the time, I didn't fully understand my own feelings, but looking back now, I realize I wanted to temporarily escape from reality. My coach and seniors beat me every day, and my parents expected too much from me, so I was under significant pressure, and I knew I wasn’t talented enough to become a professional athlete. Since I couldn't say that I would quit, I think things kept building up in my heart. Then, I ran away from home one morning, saying I was headed to training. (An, 28, female/basketball)

Considering how much money my parents had invested in my training, I knew I shouldn’t have done that. The coaches weren't teaching us anything, they just made us throw balls and hit them. I didn't run away because I didn't enjoy sports; I was frustrated—frustrated beyond words. I felt stuck, as if I belonged nowhere, and at that age, I couldn't stand the reality that even if I won a competition, I couldn't make it to the team competition because of athletes who are not as talented as I am but who coaches support because of bribes or same-school ties. (Han, 29, female/tennis)

I ran away to the countryside, but after a week, I ran out of money and had nothing to do, so I returned home on my own. Still, I wanted to express it even if it was in that way. I wanted to quit, but my parents were expecting too much of me, and I felt like I was going crazy. (Park, 28, male/baseball).

In fact, when student athletes try to run away from home, they are mostly aware of its not being a solution. The longer they stay away, the more guilty they feel for failing to repay their parents’ sacrifices, and the more frustration builds upon return. However, the study participants had in common a desperation and a need to escape from their situation by any means. Of course, the primary reason for being cornered in such a desolate situation is not the poor performance of the athlete but the university entrance structure, wherein 99% of student athletes cannot help but fall into such a hopeless (30, 113, 149). Furthermore, preconceptions regarding student athletes cannot be dismissed as a matter of athletes' responsibility alone.

Beyond familial conflicts, the rigid university entrance structure and societal expectations further exacerbate these issues, making it nearly impossible for student athletes to consider alternative career paths (42, 106, 130, 138, 141, 142, 186, 187). The belief that athletes must focus only on sports, along with weak career support, traps them in a narrow path with few alternatives.

I really hated it. I couldn't even bear to glance at a soccer ball, and I didn't want to go home. When I saw my parents, every time I saw my parents, I felt overwhelmed with guilt for failing as an athlete. But instead of expressing that guilt, I lashed out and said hurtful things … I did manage to tell them once that I wanted to quit, but when I saw my mother break down in tears, I couldn't bring myself to bring it up again. (Seong, 24, male/baseball)

In many countries abroad, systems exist that enable student-athletes to balance sports, exercise, and academics simultaneously (168, 169), but in Korea, only the law, School Sports Promotion Act (170), stipulates the continuation of their studies, and there is a prevailing societal expectation that student-athletes should focus exclusively on sports, often at the expense of their academic development (3, 40–42).

In other countries, there are athletes who excel academically while also competing at the national level. It is difficult to understand why Korea cannot adopt a similar system. If I had been born in a different country, my situation would undoubtedly have been completely different. (Kang, 22, female/tennis)

Running away can be seen as a form of non-verbal resistance. It reflects deep emotional distress and a desire to break away from oppressive circumstances. Therefore, in order to eliminate the negative influence of this extreme filial piety culture, while changing the perceptions of student athletes and parents is the top priority (42, 166), there is also a need to change the mindset of equating wealth and honour with success. This also applies to the hierarchical structure of high school graduation < junior colleges < local universities < metropolitan universities (42, 97, 98) that promotes unfair perceptions in society.

Through the interviews in this study, the common perception of success among student athletes can be summarized as the pursuit of money and fame. Achieving success as an athlete in Korea and globally is an extremely rare event, with only a small percentage of individuals reaching the top while the majority do not.

One does not necessarily have to make high earnings and become famous in order to succeed. This deeply ingrained belief in equating success with fame and wealth stems from past generations' experiences, where financial security was the only measure of success (42, 83, 96–98). The economic hardships that many parents faced in their youth have led them to prioritize stability and prestige over personal fulfillment (171, 183). However, as societal values evolve, redefining success to include personal fulfillment and happiness becomes crucial. Demonstrating to one's parents that living humbly, raising a family, and being happy can also be seen as success shows a shift in the perception of filial piety culture in Korea (172). This redefinition of success is also significant in the context of sports parenting. In Korean society, many parents have traditionally equated the success of their children who are athletes with fame and financial achievement (83, 101, 173). However, encouraging student-athletes to explore their aptitudes, interests, and diverse career opportunities beyond their athletic performance reflects a broader shift toward a more sustainable and fulfilling concept of success (161, 173, 174).

## 4 International and cross-cultural implications

Our findings on extreme filial pressure among Korean student-athletes reflect similar dynamics in other Confucian societies—such as China, Taiwan, Singapore, Hong Kong, and Japan—where family honour often overrides individual choice (175, 176). In these contexts, academic or athletic success is viewed as repayment of parental sacrifice, and authoritarian expectations can undermine youth autonomy (63). To mitigate these pressures, policymakers should implement dual-career frameworks—like the EU's flexible-curriculum and credit recognition models—that allow athletes to continue their education alongside intensive training (133, 177, 178). Simultaneously, parent-education programs grounded in research on autonomy-supportive vs. controlling parenting (179, 180) can teach caregivers to praise effort, provide structure, and encourage decision-making rather than impose undue pressure.

Ultimately, culturally sensitive approaches are needed to balance family values with youth autonomy. For example, guidance and education programs could help students develop communication and negotiation skills, allowing them to honor parental expectations while still pursuing their own goals. In doing so, practitioners might draw on Bedford and Yeh's (181) dual filial piety model, which emphasizes the value of gratitude and mutual care over authoritarian obligation. These strategies can help families uphold traditional values while empowering young athletes to thrive both emotionally and independently.

## 5 Conclusions

This study examined how extreme filial piety culture affects student-athletes in Korean school sports departments and the consequent career strategies of student-athletes and their parents. To achieve this, in-depth interviews were conducted with nine primary study participants and one auxiliary participant. Based on the collected data, the following key conclusions were drawn.

First, student-athletes often withdraw from sports or forgo university enrollment due to financial constraints and the pressure to alleviate their parents' burdens. This “Athlete Melt” phenomenon shows how students see their careers as obligations—not choices—due to structural problems in Korea's sports system.

Second, the expectation of securing a prestigious university placement through athletic performance remains a dominant factor shaping student-athletes' career trajectories. However, this process often involves non-transparent recruitment practices, such as “tie-in sales”, where student-athletes comply with career paths dictated by external pressures, leading to long-term psychological distress and reduced self-esteem.

Third, the decision to continue or withdraw from sports is often hindered by feelings of guilt and indebtedness toward parents. Many student-athletes hesitate to pursue alternative career paths, fearing they may fail to meet societal and familial expectations. As a result, some resort to drastic decisions such as running away from home or persisting in an unsustainable athletic career until reaching a dead end.

These findings show that extreme filial piety in Korea strongly shapes how student-athletes make career decisions. The perceived necessity to reciprocate parental sacrifices through athletic success contributes to structural barriers, limiting students' autonomy in career selection. Furthermore, the lack of institutional support for dual-career pathways exacerbates the challenges faced by student-athletes navigating between academics and sports.

## 6 Recommendations

Future studies should aim to prevent dropout and the “Athlete Melt” phenomenon by reorganizing policies to create an environment that empowers student-athletes to make autonomous career decisions. Establishing comprehensive support systems to guide both post-athletic career development and the fostering of dual careers is essential. Practically, this involves offering flexible class schedules, vocational training, and career counseling so athletes can balance school and sports. Moreover, reducing the undue influence of high school and college coaches on recruitment and career transitions is critical. In Korean sports, coaches often serve as intermediaries between players, their parents, and higher education or professional institutions, which can lead to non-transparent practices such as tie-in sales. Shifting the control of recruitment and contractual matters directly to institutions or designated representatives may help prevent these practices. Long-term research should support a social system where studying is seen as normal for athletes and dual careers are encouraged. Finally, altering entrenched perceptions about the supremacy of athletic victory, rigid university hierarchies, and extreme filial piety through targeted media initiatives is vital for fostering a balanced model of familial responsibility.

## Data Availability

The original contributions presented in the study are included in the article, further inquiries can be directed to the corresponding author.

## Footnotes

^1^This term adapts the U.S. concept of "summer melt." In the Korean context, it refers to student-athletes who refrain from university enrollment due to structural barriers, financial pressures, and societal expectations—even after years of athletic dedication.

^2^Introduced in 1972, the sports specialist admission system enabled student-athletes to enter universities based purely on athletic ability. The goal was to foster elite athletes and enhance national prestige. However, this policy led to an education system where academic achievement was largely disregarded. The limitations of this approach became evident in 2016 when an equestrian athlete was found to have gained university admission through irregular means. In response, the Ministry of Education implemented a ‘minimum level of education’ requirement for student-athletes in 2017, though its effectiveness remains limited.

^3^In Korea, when a student drops out of high school, their GPA, a key factor in college admissions, is erased. University admissions are divided into early and regular processes: early admission prioritizes GPA with a minimum KSAT requirement, while regular admission relies more on KSAT scores. Student-athletes often neglect academics, making both routes difficult. Early admission excludes them due to low GPAs, and regular admission penalizes weak academic performance. To overcome this, some quit sports and drop out to reset their GPA. Once their GPA is erased, it can be replaced by a recalculated KSAT score. This allows them to reapply under better conditions. This makes dropping out a strategic move for entering prestigious universities.
